# *Marine Drugs* Best Paper Award 2014

**DOI:** 10.3390/md12021157

**Published:** 2014-02-21

**Authors:** Alejandro M. S. Mayer

**Affiliations:** *Editor-in-Chief*, Department of Pharmacology, CCOM, Midwestern University, 555 31st. Street, Downers Grove, Illinois 60515, USA; E-Mail: amayer@midwestern.edu

*Marine Drugs* initiated a “Best Paper Award” in 2013 [1] to recognize outstanding papers published in our journal in the area of research, development and production of drugs from the sea, including marine natural product chemistry. We are pleased to announce the second “*Marine Drugs* Best Paper Award” for 2014. Nominations were selected by the Editor-in-Chief, Associate Editors and Editorial Board Members of *Marine Drugs* from all papers published in 2010; reviews and articles being evaluated separately. The following three papers have won this award:

**Article Award:**

*First Prize*

Sheila Marie Pimentel-Elardo*, Svitlana Kozytska, Tim S. Bugni, Chris M. Ireland, Heidrun Moll and Ute Hentschel

Anti-Parasitic Compounds from *Streptomyces* sp. Strains Isolated from Mediterranean Sponges

*Mar. Drugs* **2010**, *8*(2), 373-380; doi:10.3390/md8020373

Available online: http://www.mdpi.com/1660-3397/8/2/373

*Second Prize*

Ruth Harris, Elena Lecumberri and Angeles Heras *

Chitosan-Genipin Microspheres for the Controlled Release of Drugs: Clarithromycin, Tramadol and Heparin

*Mar. Drugs* **2010**, *8*(6), 1750-1762; doi:10.3390/md8061750

Available online: http://www.mdpi.com/1660-3397/8/6/1750

**Review Award:**

*First Prize*

Leanne Pearson, Troco Mihali, Michelle Moffitt, Ralf Kellmann and Brett Neilan *

On the Chemistry, Toxicology and Genetics of the Cyanobacterial Toxins, Microcystin, Nodularin, Saxitoxin and Cylindrospermopsin

*Mar. Drugs* **2010**, *8*(5), 1650-1680; doi:10.3390/md8051650

Available online: http://www.mdpi.com/1660-3397/8/5/1650

Prize Awarding Committee members highly recommend the article “Anti-Parasitic Compounds from *Streptomyces* sp. Strains Isolated from Mediterranean Sponges”, and commented “Search for novel antiparasitic drugs are of world-wide concern. The paper has the merit of highlighting the still overlooked potential of actynomicete-derived metabolites. In addition, it also exemplifies how investigation on already known drugs can reveal unexpected pharmacological activities. In this case, well known antibiotics have revealed activity against tropical diseases, for which there is urgent need of viable alternatives to current therapeutic options.”

For the review: “On the Chemistry, Toxicology and Genetics of the Cyanobacterial Toxins, Microcystin, Nodularin, Saxitoxin and Cylindrospermopsin”, they said “Cyanobacterial toxins are of global concern and climate change will enhance cyanobacterial blooms and presence of toxins in the environment. This review on cyanobacterial toxins fulfills the needs of providing integrated updated information on chemistry, toxicology and genetics of this fascinating class of secondary metabolites.”

We believe that these three papers are valuable contributions to *Marine Drugs* and marine drug discovery research. On behalf of the Prize Awarding Committee and the Editorial Board of *Marine Drugs*, we would like to congratulate these three teams for their excellent work. In recognition of their accomplishment, Dr. Sheila Marie Pimentel-Elardo and Dr. Angeles Heras will receive a prize of 600 CHF and 400 CHF, respectively, and the privilege to publish an additional paper free of charge in open access format in *Marine Drugs*, after the usual peer-review procedure. Dr. Brett Neilan will also be awarded the privilege of publishing an additional research paper free of charge in open access format in *Marine Drugs*, after the usual peer-review procedure.

*Prize Awarding Committee*

*Editor-in-Chief*

**Prof. Dr. Alejandro M. S. Mayer**

Department of Pharmacology, CCOM, Midwestern University, 555 31st. Street, Downers Grove, Illinois 60515, USA

E-Mail: amayer@midwestern.edu

*Associate Editor*

**Prof. Dr. Jordan K. Zjawiony**

Department of Pharmacognosy, School of Pharmacy, University of Mississippi, MS 38677, USA

E-Mail: jordan@olemiss.edu

*Associate Editor*

**Prof. Dr. Nobuhiro Fusetani**

Graduate School of Fisheries Sciences, Hokkaido University, 3-1-1 Minato-cho, Hakodate 041-8611, Japan

E-Mail: anobu@fish.hokudai.ac.jp

*Editorial Board Member*

**Prof. Dr. Orazio Taglialatela-Scafati**

Dipartimento di Chimica delle Sostanze Naturali, Università di Napoli Federico II, Via Montesano 49, I-80131 Napoli, Italy

E-Mail: orazio.taglialatela@unina.it

*Editorial Board Member*

**Prof. Dr. Vassilios Roussis**

University of Athens, School of Pharmacy, Department of Pharmacognosy and Chemistry of Natural Products, Panepistimiopolis Zografou, GR 15771, Athens, Greece

E-Mail: roussis@pharm.uoa.gr
